# Bacteremia Prevention during Periodontal Treatment—An In Vivo Feasibility Study

**DOI:** 10.3390/antibiotics12101555

**Published:** 2023-10-20

**Authors:** Patrick Jansen, Georg Conrads, Johannes-Simon Wenzler, Felix Krause, Andreas Braun

**Affiliations:** 1 Clinic for Operative Dentistry, Periodontology and Preventive Dentistry, Rheinisch-Westfälische Technische Hochschule University Hospital, Pauwelsstrasse 30, 52074 Aachen, Germany; jwenzler@ukaachen.de (J.-S.W.); fkrause@ukaachen.de (F.K.); anbraun@ukaachen.de (A.B.); 2 Division of Oral Microbiology and Immunology, Clinic for Operative Dentistry, Periodontology and Preventive Dentistry, Rheinisch-Westfälische Technische Hochschule University Hospital, 52074 Aachen, Germany; gconrads@ukaachen.de

**Keywords:** periodontal treatment, periodontal therapy, nonsurgical periodontal therapy, bacteremia, laser, laser disinfection

## Abstract

The link between periodontitis and systemic diseases has increasingly become a focus of research in recent years. In this context, it is reasonable—especially in vulnerable patient groups—to minimize bacteremia during periodontal treatment. The aim of the present in vivo feasibility study was to investigate the possibility of laser-based bacteremia prevention. Patients with stage III, grade B generalized periodontitis were therefore treated in a split-mouth design either with prior 445 nm laser irradiation before nonsurgical periodontal therapy or without. During the treatments, clinical (periodontal measures, pain sensation, and body temperature), microbiological (sulcus samples and blood cultures before, 25 min after the start, and 10 min after the end of treatment), and immunological parameters (CRP, IL-6, and TNF-α) were obtained. It was shown that periodontal treatment-related bacteremia was detectable in both patients with the study design used. The species isolated were *Schaalia georgiae*, *Granulicatella adiacens*, and *Parvimonas micra*. The immunological parameters increased only slightly and occasionally. In the laser-assisted treatments, all blood cultures remained negative, demonstrating treatment-related bacteremia prevention. Within the limitations of this feasibility study, it can be concluded that prior laser disinfection can reduce bacteremia risk during periodontal therapy. Follow-up studies with larger patient numbers are needed to further investigate this effect, using the study design presented here.

## 1. Introduction

Periodontitis is an inflammatory disease of all structures of the periodontium, maintained by microorganisms in the subgingival biofilm. Inflammatory changes can lead to loss of bone, collagen, and attachment. In advanced disease and without adequate treatment, the consequence may be loss of the affected teeth. Irreversible periodontal damage is usually preceded by reversible gingivitis due to dysbiotic microbial colonization of the tooth and/or gum. 

In addition to this basic condition, systemic diseases represent cofactors for periodontitis, and some of these may in turn be influenced by the periodontitis itself. These systemic diseases include, for example, diabetes mellitus, cardiovascular disease (such as coronary artery disease), and prematurity [1,2,3].

In particular, the bidirectionality between diabetes mellitus and periodontitis has been well studied. Various clinical studies have shown that periodontitis is more common in diabetics than in non-diabetics [4,5,6]. Periodontitis, in turn, may also influence diabetes mellitus. For example, the risk of diabetics dying from coronary heart disease or nephropathy is significantly higher if they have advanced periodontitis [7]. 

In addition to diabetes, many studies have also shown an association between periodontitis and coronary heart disease [2,8,9]. 

Recently, in addition to the systemic diseases already mentioned, a link between periodontitis and Alzheimer’s disease (AD) has also been discussed [10,11,12,13]. A prospective clinical study showed that active, chronic periodontitis was associated with a decline in mental abilities in nursing home residents [14]. In particular, *Porphyromonas gingivalis* (*P. gingivalis*), one of the main pathobionts involved in periodontitis, appears to play a key role in this regard [11,15,16]. Thus, it is clear from these examples that periodontal health is closely related to systemic health.

Removal of the soft biofilm or mineralized deposits adhering to the tooth surface and thus the removal of periodontal pathogenic microorganisms (pathobionts) by scaling and root planing (SRP) is one of the main objectives of nonsurgical periodontal therapy and supportive periodontal therapy [17], regardless of the systemic factors that influence the event. Conventional methods such as the use of manual or ultrasound instruments are available for this purpose, and their effectiveness is considered to be comparable [18,19]. Another option is the use of air polishing systems or laser systems for cleaning affected tooth surfaces [20,21,22,23]. However, the benefit of root surface cleaning in periodontal therapy, e.g., with the Er:YAG laser, remains questionable due to the high heterogeneity of clinical studies [24]. Generally, it is possible to achieve a reduction in periodontal pathobionts using the various methods of surface instrumentation [25,26]. It cannot be assumed that complete removal of pathogens is achieved. Even the additional local application of an antibiotic—for example, using tetracycline-releasing sutures [27] or doxycycline-releasing gels [28,29]—cannot guarantee that all pathogens will be permanently removed. Furthermore, especially in advanced cases with high probing depths (≥6 mm), furcation involvement or, for example, root concavities, it becomes difficult or even impossible to perform this cleaning successfully through nonsurgical approaches alone; here, it then makes sense to consider surgical approaches (resective or regenerative) [30].

During such cleaning of the tooth or root surfaces, which is an invasive procedure, there is a risk of bacteremia [31,32,33]. In most cases, this bacteremia has no (or unnoticed) clinical relevance in the majority of patients. However, such bacteremia can cause dangerous infections in patients who have heart valve replacements or are immunosuppressed. Here, antibiotic prophylaxis and/or removal of the pathobionts makes all the more sense [34]. A direct effect of these bacteremia during nonsurgical periodontal therapy on other systemic diseases, as mentioned above, has not yet been proven. However, this topic itself should continue to be critically observed scientifically. 

Another effective approach to the elimination of pathobionts could be the use of light energy, in the form of lasers. Diode lasers achieve particularly good results in adjunctive periodontal therapy [35,36]. They also appear to lead to the effective elimination of periodontal microorganisms. One study showed, in a split-mouth design, that the use of a diode laser (810 nm) before ultrasound scaling in patients with gingivitis resulted in a significant reduction in the prevalence of odontogenic bacteremia [37]. 

The severity of periodontal disease can also be exacerbated by specific genotypes that influence the immune response, with a well-known example being the interleukin-1 polymorphism. Likewise, periodontitis may start significantly earlier in such patients than in patients without this polymorphism [38]. In addition to the bactericidal effects of lasers, it is also plausible to consider photobiomodulatory effects, especially with blue and green laser light, which may have a positive influence on the immune response and post operative inflammation and wound healing [39]. A current approach in this context involves the use of a novel diode laser with a wavelength of 445 nm. In an in vitro study, this wavelength was shown to effectively reduce a broad spectrum of pathobionts, including *Aggregatibacter actinomycetemcomitans*, *P. gingivalis*, *Staphylococcus aureus*, and *Candida albicans*, with a relatively short application time and low total energy [40]. This approach needs to be investigated further. The aim of the present feasibility study was therefore to evaluate the bacteremia-preventive effect of disinfection with a diode laser before conventional nonsurgical periodontal therapy—testing the hypothesis that a bacteremia-preventive effect of laser irradiation prior to periodontal treatment can be demonstrated with the study design used.

## 2. Results

The present feasibility study demonstrated that the study design is capable of detecting pathobionts that had entered the bloodstream due to therapy-induced bacteremia. The laser parameters used also demonstrated a bacteremia-preventive effect.

### 2.1. Clinical Parameters

#### 2.1.1. Patient 1

The patient had stage III, grade B generalized periodontitis. Probing pocket depths of up to 6 mm were present, the highest degree of mobility was I (teeth 25 and 45), and the highest degree of furcation involvement was also I (teeth 17, 16, 26, 37, and 36). Periodontal recessions were mainly present in the mandibular anterior region, but these were only slight (recession type I: maximum 1 mm).

At the first appointment, the patient had a score of 0 on the visual analog scale (scores of 0–10) for laser treatment (inactive laser) and a score of 6 for subsequent SRP. At the second appointment, the scores were 3 on the visual analog scale for laser treatment (active laser) and 4 for subsequent SRP.

Measurement of the patient’s body temperature (in the ear) showed the following values at the first appointment: before treatment: 36.6 °C; after treatment: 36.6 °C. Measurement of the patient’s body temperature showed the following values at the second appointment: before treatment: 35.9 °C; after treatment: 36.5 °C.

#### 2.1.2. Patient 2

The patient had stage III, grade B generalized periodontitis. Probing pocket depths of up to 10 mm (at one tooth) were present, the highest degree of mobility was I (teeth 17, 15, 14, 12–22, 25, 27, 37, 35, 31, and 41), and the highest degree of furcation involvement was II (teeth 17, 16, 27, and 46; degree I: 14, 24, 37, 36, and 47). Periodontal recessions were mainly present in the mandibular anterior region, but these were only slight (recession type I: maximum 3 mm).

At the first appointment, the patient had a score of 0 on the visual analog scale (scores of 0–10) for laser treatment (inactive laser) and a score of 2 for subsequent SRP. At the second appointment, the scores were 2 on the visual analog scale for laser treatment (avtive laser) and 2 for subsequent SRP.

Measurement of the patient’s body temperature (in the ear) showed the following values at the first appointment: before treatment: 36.4 °C; after treatment: 36.1 °C. Measurement of the patient’s body temperature showed the following values at the second appointment: before treatment: 36.1 °C; after treatment: 35.6 °C.

### 2.2. Microbiological and Immunological Parameters

#### 2.2.1. Patient 1

Sulcus sampling identified various bacteria at the baseline examination, including *Schaalia* (formerly *Actinomyces*) *georgiae*, which was also detected in the anaerobic blood culture sample during SRP at the first appointment (see below). The complete list at each examination time point is shown in Table 1.

At the first appointment, the results of the aerobic and anaerobic blood culture analyses were as follows. The baseline examination did not reveal any anomalies or detectable transient bacteremia. In the samples taken 25 min after the start of SRP, *Granulicatella adiacens* was detected in the aerobic blood culture after several days of incubation and *S. georgiae* in the anaerobic blood culture. Samples taken 10 min after the completion of SRP did not show any anomalies, as in the baseline examination. Aerobic and anerobic blood culture analyses at the second appointment were unremarkable at the baseline examination, as well as at examinations 25 min after the start of SRP and 10 min after completion of SRP. Thus, no transient bacteremia was detected at this appointment.

**Figure 1:**
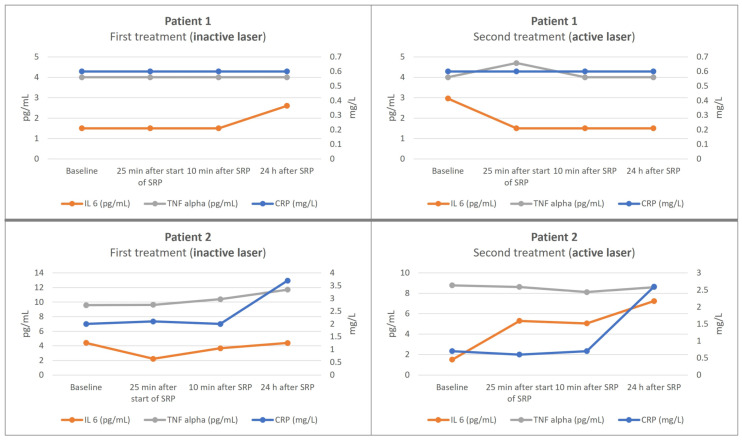
Diagrams of the kinetics of the inflammatory parameters (detection thresholds: IL 6: 1.5 pg/mL; TNF alpha: 4.0 pg/mL; CRP: 0.6 mg/L).

Immunological parameters—C-reactive protein (CRP) reference range < 5.0 mg/L, interleukin-6 (IL-6) reference range < 7.0 pg/mL, and tumor necrosis factor alpha (TNF-α) reference range < 8.1 pg/mL—showed the following courses in patient 1 at the first appointment: there were no changes at baseline, 25 min after the start of SRP, or 10 min after the end. One day after the first SRP, there was only a change in IL-6 (2.6 pg/mL, compared to <1.5 pg/mL at the baseline examination). The immunological parameters showed the following courses at the second appointment: at the baseline examination, the value for IL-6 was 2.96 pg/mL; at the subsequent examinations, this value decreased again to <1.5 pg/mL. The values for TNF-α were <4.0 pg/mL at the baseline examination, 4.7 pg/mL at the examination 25 min after the start of SRP, and <4.0 pg/mL again at the subsequent examinations. The values for CRP did not show any changes at the different time points. The kinetics of the inflammatory parameters are visually presented in Figure 1. 

#### 2.2.2. Patient 2

Sulcus sampling identified various bacteria at the baseline examination, including *Parvimonas micra*, which was also detected in the anaerobic blood culture sample during SRP at the first appointment (see above). *P. gingivalis* was detected in the sulcus sample at baseline, but not in the blood cultures. The complete list at each examination time point is shown in Table 1.

At the first appointment, the results of the aerobic and anaerobic blood culture analyses were as follows. The baseline examination did not reveal any anomalies or detectable transient bacteremia. In the samples taken 25 min after the start of SRP, *P. micra* was detected in the anaerobic blood culture after several days of incubation. Samples taken 10 min after the completion of SRP did not show any anomalies, as in the baseline examination. Aerobic and anerobic blood culture analyses at the second appointment were unremarkable at the baseline examination, as well as at examinations 25 min after the start of SRP and 10 min after the completion of SRP. Thus, no transient bacteremia was detected at this appointment.

Immunological parameters (CRP, IL-6, TNF-α) showed the following courses in patient 2 at the first appointment: CRP showed a slight increase 24 h after treatment (baseline: 2 mg/L; 24 h after treatment: 3.7 mg/L), but IL-6 remained more or less stable. TNF-α was already elevated above the reference range before the start of treatment, this did not change during and after treatment. At the second appointment, CRP showed a similar course (baseline: 0.7 mg/L; 24 h after treatment: 2.6 mg/L). IL-6 showed a slight increase above the reference range 24 h after treatment (7.25 pg/mL). TNF-α was elevated above the reference range before treatment, as at the first appointment, and again, this did not change during and after treatment. The kinetics of the inflammatory parameters are visually presented in Figure 1.

## 3. Discussion

The present feasibility study demonstrated that the use of a diode laser with a wavelength of 445 nm has potential bacteremia-preventive effects when applied immediately before SRP. However, it must be clearly stated that this effect needs to be further investigated and proven in follow-up studies with larger numbers of patients. During periodontal treatment, transient bacteremia inevitably occurs. The incidence of bacteremia depends on the degree of periodontal inflammation [31]. Kinane et al. detected bacteremia in 13% of patients after ultrasound scaling and in 20% after periodontal probing [33]. In comparison with the present feasibility study, the blood samples in that study were only taken after the periodontal procedures, so not all bacteremia may have been detected due to phagocytosis of the bacteria by immune cells. In another study, bacteremia was detected in 73.8% of patients immediately after SRP, and 19% of the blood cultures were still positive 30 min after the completion of SRP [41]. Zhang et al. detected bacteremia in 33.3% of patients 5 min after the start of SRP [42]. These studies all detected bacteremia, but with marked differences in prevalence. This may be mainly due to the different methods used and different collection times [32]. To avoid missing bacteremia as much as possible, the collection times for blood cultures in the present study design were adjusted to follow the schedule described by Beutler et al. [43]. In contrast with most studies, the present feasibility study not only investigated the presence of bacteria in blood, but also took sulcus samples to demonstrate that the blood isolates actually originated from the periodontal pockets.

It has been shown that cleaning all quadrants of a dentition within 24 h resulted in an increased acute-phase response, in comparison to cleaning quadrants in individual sessions with a time interval of 1 week between each session [44]. However, periodontal microorganisms can enter the bloodstream not only during periodontal therapy, but also as a result of professional mechanical plaque removal, or through micro-injuries from mere chewing or tooth-brushing, in accordance with the rule that the more severe the periodontitis, the more severe the bacteremia [31,43,45]. In most cases, this is assumed to have no clinical consequences (or only unrecognized ones), but in vulnerable patient groups such as patients with heart valve replacements, there may be serious sequelae. One potential sequela is infective endocarditis, which should be avoided in such cases using antibiotic prophylaxis [46]. Clear disadvantages of systemic antibiotic prophylaxis are its effects on the whole body, including the commensal microbiome and increasing resistance rates. Employing a laser device, we do not assume that complete sterilization of the periodontal pocket is possible, but we hypothesize that at least such a temporary bacterial reduction in pockets is possible with laser disinfection through which bacteremia can be avoided.

In addition to the administration of systemic antibiotics to reduce or prevent transient bacteremia, local disinfection methods may also be an option. These include, for example, mouth rinses. However, a single mouth rinse containing chlorhexidine before scaling did not show any effect on bacteremia reduction in one study [47]. In addition to oral rinses, local laser administration may also be considered. In a clinical study, a diode laser with a wavelength of 810 nm was used before ultrasonic scaling in 22 patients with gingivitis, also with a split-mouth research design. Bacteremia was detected in 15 patients after ultrasound scaling alone, and after laser treatment in only eight patients (810 nm; flexible fiber optic 300 µm; repeated beam: 0.2 s on and 0.3 s off; output power 1.0 W; laser application duration for each tooth: 15 s) before ultrasound scaling [37].

In the present study, a laser wavelength that is effective against a broad spectrum of pathobionts—including *A. actinomycetemcomitans*, *P. gingivalis*, *S. aureus*, und *C. albicans*—was used for a bacteremia reduction approach. The wavelength used was 445 nm at a power of 0.5 W, and the laser was used in continuous-wave mode with a 320 µm fiber for 1 min for each tooth. In a pilot study, these parameters showed significant reductions in *P. gingivalis* in comparison with a control group in vitro [40]. This wavelength has a very high absorption level in the spectrum of hemoglobin and pigmented bacteria, which may explain the strong reduction in *P. gingivalis.* As this was a feasibility study and data were generated from only two patients, *P. gingivalis* was detected in sulcus samples in only one patient. The bacteremia-preventive properties of the laser treatment studied here might be of interest not only for vulnerable patient groups, but also for healthy patients. As mentioned above, periodontitis and Alzheimer’s disease have recently been associated. In particular, lipopolysaccharides of *P. gingivalis* have been detected in the brains of deceased patients with Alzheimer’s disease [48]. The laser treatment described here might be suitable for avoiding or at least reducing this type of bacterial migration as a result of periodontal therapy, thus potentially reducing the risk of systemic diseases.

As mentioned above, Graziani et al. demonstrated that the acute-phase response was greater in full-mouth nonsurgical therapy than in quadrant nonsurgical therapy [44]. Whether the same difference existed between the two experimental groups represented in the present study was also investigated. Unfortunately, due to the small number of patients, no clear trends can be demonstrated. In patient 1, IL-6 increased slightly 24 h after conventional treatment, but this was not detected 24 h after the appointment involving laser treatment; however, there was a transient increase in TNF-α 25 min after the start of SRP, which decreased again in the subsequent examinations. In patient 2, their TNF-α levels were already elevated at baseline and did not change during the course of the examinations on both dates. The other parameters also did not show any clear trends. Follow-up studies with larger numbers of patients may clarify whether laser disinfection with the corresponding reduction in, or prevention of, bacteremia is also associated with a lower acute-phase response.

Overall, despite the small number of patients and the limitations of this feasibility study, it can be concluded that the study protocol is capable of detecting bacteremia after periodontal therapy and that the laser disinfection method described could have potential bacteremia-reducing or even bacteremia-preventive properties. This needs to be confirmed in follow-up studies, which are currently underway.

## 4. Materials and Methods

### 4.1. Clinical Parameters

As a preparatory measure, situation models of both jaws in each patient were made in order to produce individual splints. These covered the occlusal surfaces and extended to the equator of all teeth. At six locations (mesio-vestibular, central vestibular, disto-vestibular, mesio-oral, central oral, and disto-oral), milled guiding grooves were used for defined insertion of a pressure-calibrated probe (Aesculap DB764R, Aesculap, Tuttlingen, Germany) into the gingival pocket. Using the individual splints produced in this way, the pocket depths, periodontal recession, clinical attachment level, and bleeding on probing were determined at the various examination time points by one investigator (P.J.). Bleeding points occurring within 30 s of probing were documented and reported as a percentage for the entire dentition. In addition, furcation involvement and the degree of looseness were recorded.

Pain sensation during treatment was assessed by the patients using a visual analog scale after the treatment session.

Body temperature was measured before and after treatment using an infrared thermometer in the ear (Thermoscan PRO 6000, Braun, Kronberg im Taunus, Germany).

### 4.2. Microbiological and Immunological Parameters

For local microbiological diagnosis, samples were taken using sterile paper points (ISO 45) in the two deepest pockets of each quadrant (eight in total) at baseline, 7 days after the first appointment/SRP, at the second appointment/SRP (14 days after the first SRP), and 7 days after the second appointment/SRP. These were then analyzed by culturing on various media (with and without the addition of sheep blood) at 37 °C in appropriate atmospheric conditions and identified by Maldi-TOF (mass spectrometry).

To be able to detect systemic bacteremia after the treatments and compare it with the baseline findings, peripheral blood (25 mL) was drawn before the start of treatment, 25 min after the start, and 10 min after the end of the treatment in both the control group and the experimental group by one investigator (J.-S.W.). The patients fasted for this purpose to ensure standardization and the better accessibility of microbiological and immunological parameters. Peripheral blood cultures were analyzed using standard operational procedures by the laboratory diagnostic center at the University Hospital in Aachen, Germany. However, the blood cultures (aerobic and anaerobic) were kept for longer (maximum 21 days) and were plated several times to avoid overlooking any slow-growing species, with special emphasis on *P. gingivalis* and other black-pigmented bacteria. It should be noted that not every bacterial cell entering the bloodstream can be detected using this (or any) method, as most are very rapidly phagocytosed and thus killed and eliminated.

In addition, the samples were analyzed for immunological parameters in blood plasma to allow conclusions to be drawn concerning the acute-phase reaction. C-reactive protein (CRP), interleukin-6 (IL-6), and tumor necrosis factor alpha (TNF-α) were of particular interest. These parameters were also monitored 24 h after the respective treatment appointments.

The blood samples mentioned above were obtained from the median cubital veins. The areas of the puncture were carefully disinfected beforehand using a skin antiseptic (Octeniderm, Schülke, Norderstedt, Germany). For this purpose, the investigator responsible for blood sampling wore sterile gloves, which he kept on for the entire blood-sampling procedure. The puncture procedure was repeated at each blood collection time point. These precautions were implemented to minimize the possibility of contamination of the blood samples with skin commensals. The blood collection procedures themselves were performed with a Safety Multifly cannula (Sarstedt, Nürnbrecht, Germany). A blood culture adapter (Sarstedt, Nürnbrecht, Germany) was used for blood cultures; the aerobic blood culture bottle (Bactec Plus Aerobic/F Culture Vials 30 mL, Becton Dickinson, Franklin Lakes, NJ, USA) was inoculated first, followed by the anaerobic blood culture bottle (Bactec Lytic/10 Anaerobic/F Culture Vials 40 mL, Becton Dickinson, Franklin Lakes, NJ, USA). A blood collection system was then used for the immunological parameters (S-Monovette, Sarstedt, Nürnbrecht, Germany). The blood culture and immunological analyses were conducted in the laboratory diagnostic center at RWTH Aachen University Hospital, and the other microbiological analyses were conducted in the Division of Oral Microbiology and Immunology, Clinic for Operative Dentistry, RWTH Aachen University.

### 4.3. Treatment Procedure

Treatments in this study were performed using a single-blinded, randomized, split-mouth design. Which quadrants received the conventional treatment (control quadrants) and which quadrants received experimental laser disinfection before SRP were selected in advance using a randomized computer-generated random table. The patients did not know which quadrants were to receive the active laser disinfection, since the control quadrants received inactive laser disinfection—where the laser tip was guided into the sulcus without being switched on. To exclude potential bias, the physicians who performed SRP and the measurements were also not aware of which quadrants were to receive the actual laser disinfection. This was ensured by having a separate practitioner performing the laser disinfection procedure (A.B.).

Before conventional pocket cleaning, the periodontal pockets in the experimental study group were disinfected with a diode laser (SiroLaser Blue, Dentsply Sirona, Bensheim, Germany). The wavelength was 445 nm and the power was 0.5 W, with the laser in continuous-wave mode (laser class 4). A 320 µm fiber was used to disinfect/clean circularly around the diseased tooth for 1 min under constant movement. The laser light used was guided into the gingival pocket with an optical fiber. The device setting of 0.5 W corresponded to an effective power of 0.48 W. Assuming a Gaussian laser beam profile, the power density was 1210 W/cm^2^ (fiber tip diameter 0.32 mm; 0.48 W output power). Due to the low power density, this laser light is not harmful to the gums or other surrounding tissue. However, laser safety goggles must be worn during the irradiation procedure to protect the eyes.

All of the teeth with periodontal disease were then treated with manual instruments (Gracey curettes, Hu-Friedy, Leimen, Germany) and a piezoelectric scaler (Vector, Dürr Dental, Bietigheim-Bissingen, Germany) with a slimline scaler tip. The treatment end point was the clinically assessed absence of concrements on the root surfaces. This was assessed by a dentist who was experienced in periodontal treatment (F.K.), who was not aware of which teeth had received active or inactive laser disinfection.

**Figure 2:**
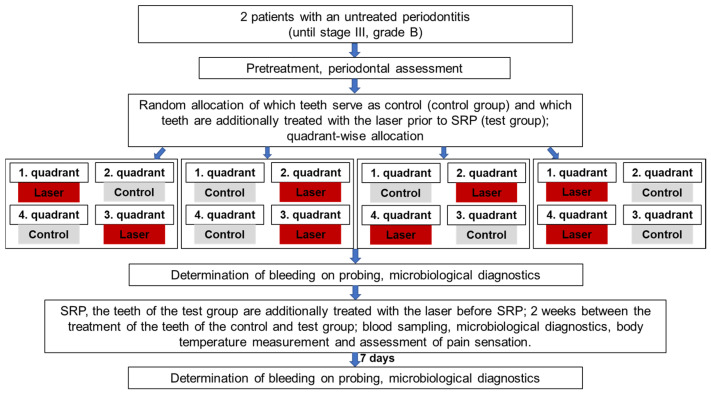
Flowchart for the study design.

Figure 2 shows the sequence of individual steps in more detail.

### 4.4. Patients

Two patients (two men, one 39 years of age and the other 63) with untreated generalized periodontitis (stage III, grade B) were recruited in the Clinic of Operative Dentistry, Periodontology, and Preventive Dentistry at Aachen University Hospital. The inclusion criteria were a minimum age of 18 years, no periodontal treatment in the previous 2 years, generalized periodontitis up to stage III, grade B, and the provision of written informed consent. The exclusion criteria were patients who had already received periodontal treatment within the previous 2 years, pregnancy, tobacco use, dental implants, metabolic diseases with possible influence on the healing process (especially diabetes), infectious diseases (such as human immunodeficiency virus or hepatitis B or C), patients undergoing chemotherapy, cystic fibrosis, and antibiotic treatment within the previous 3 months. All patients received detailed information regarding the conduction of the study and data protection. Each of the participants gave written informed consent to participate in the study. In the test plan of the present study submitted to the local ethics committee, particular attention was paid to benefit–risk assessment, the additional study-related measures (blood sampling and laser disinfection), and the use of the laser within its approval. The local ethics committee of the RWTH Aachen University Hospital saw/sees no concerns regarding the research project from an ethical and professional perspective. The study was conducted in full accordance with local and global ethical guidelines (World Medical Association Declaration of Helsinki, version X, 2013) and approved by the local ethics committee (reference number: EK 197/21; date of approval 17 August 2021).

### 4.5. Statistical Analysis

In this feasibility study, descriptive statistical analysis methods were mainly used for the two patients included. Null hypothesis significance testing is not appropriate unless the sample size is properly powered [49].

## 5. Conclusions

This feasibility study showed that the study protocol appears to be suitable for detecting bacteremia during periodontal therapy. Initial indications of a bacteremia-reducing or even bacteremia-preventive effect of the laser disinfection method described were observed. However, due to the small number of patients, this effect must continue to be investigated and proven in follow-up studies with larger numbers of patients. 

## Figures and Tables

**Table 1:** Results of the blood culture sampling and microbiological sulcus samples.

Patient 1	Time Point	Blood Culture	Assessment	Sulcus Samples (Baseline)
First treatment (inactive laser)	Baseline	Aerobic	Negative	Dominant cultivable species: *Actinomyces israelii* *Actinomyces oris* *Capnocytophaga gingivalis* *Fusobacterium canifelinum* *Parvimonas micra* *Prevotella denticola* *Prevotella nigrescens* ***Schaalia georgiae*** *Streptococcus intermedius* *Streptococcus oralis* *Veillonella parvula*
		Anaerobic	Negative	
	25 min after the start of SRP	Aerobic	*Granulicatella adiacens*	
		Anaerobic	***Schaalia georgiae***	
	10 min after SRP	Aerobic	Negative	
		Anaerobic	Negative	
Second treatment (active laser)	Baseline	Aerobic	Negative	
		Anaerobic	Negative	
	25 min after the start of SRP	Aerobic	Negative	
		Anaerobic	Negative	
	10 min after SRP	Aerobic	Negative	
		Anaerobic	Negative	
**Patient 2**	**Time point**	**Blood culture**	**Assessment**	**Sulcus samples (baseline)**
First treatment (inactive laser)	Baseline	Aerobic	Negative	Dominant cultivable species: *Actinomyces denticolens* *Actinomyces oris* *Actinomyces meyeri* *Eikenella corrodens* *Eubacterium brachy* *Fusobacterium naviforme* *Fusobacterium nucleatum* *Gemella morbillorum* ***Parvimonas micra*** *Porphyromonas gingivalis* *Prevotella denticola* *Prevotella intermedia* *Slackia exigua* *Streptococcus anginosus* *Streptococcus constellatus* *Streptococcus oralis* *Streptococcus sanguinis*
		Anaerobic	Negative	
	25 min after the start of SRP	Aerobic	Negative	
		Anaerobic	***Parvimonas micra***	
	10 min after SRP	Aerobic	Negative	
		Anaerobic	Negative	
Second treatment (active laser)	Baseline	Aerobic	Negative	
		Anaerobic	Negative	
	25 min after the start of SRP	Aerobic	Negative	
		Anaerobic	Negative	
	10 min after SRP	Aerobic	Negative	
		Anaerobic	Negative	

SRP, scaling and root planing. The bacteria names in bold refer to the bacteria that were detected both in the sulcus and later in the blood samples.

## Data Availability

The data are not publicly available due to ethical restrictions regarding patient data.
